# A microbial ecosystem enhanced by regulating soil carbon and nitrogen balance using biochar and nitrogen fertiliser five years after application

**DOI:** 10.1038/s41598-023-49140-y

**Published:** 2023-12-14

**Authors:** Fanhao Meng, Hengshan Yang, Xiuyan Fan, Xin Gao, Jicheng Tai, Rula Sa, Xuanliang Ge, Xuezhen Yang, Qingyi Liu

**Affiliations:** 1 College of Agronomy, Inner Mongolia Minzu University, Tongliao, 028000 China; 2 Inner Mongolia Autonomous Region Feed Crop Engineering Technology Research Center, Inner Mongolia Minzu University, Tongliao, 028000 China; 3 Keerqin Zuoyizhong Banner Agricultural Technology Extension Center, Tongliao, 028000 China

**Keywords:** Microbiology, Plant sciences

## Abstract

The indiscriminate use of nitrogen fertiliser (NF) is a obstruction to improve soil quality and crop yields. However, the effect of biochar and NF on soil microbial ecosystem (SME) and crop yields is unknown. A five-year field experiment in China aimed to evaluate the effects of biochar and nitrogen fertiliser (NF) combination on soil structure, C-to-N ratio (CNR), microbial biomass, and spring maize yield. Biochar and NF were applied at different rates, and the combined application resulted in a soil solid–liquid–gas ratio closer to the ideal value. The use of biochar alone and in combination with NF significantly increased soil's C, N, and CNR. A moderate application of biochar and NF resulted in favourable biological and chemical properties of the soil. The application of biochar and NF at moderate levels led to an increase in SME, with the B8N150 producing the highest yield. The highest yield of B8N150 represents a 24.25% increase compared to the unfertilized control and a 9.04% increase compared to B0N150. Moderate use of biochar and NF could be beneficial in areas with similar climatic conditions.

## Introduction

Rapid growth in global population and accelerating urbanisation have led to a significant reduction in arable land. The improvement of arable land quality and the assurance of food security are major concerns in modern agriculture. However, current agricultural practices often prioritised high crop yields, and leading to the excessive use of chemical fertilisers, especially nitrogen fertiliser (NF). This indiscriminate use of NF has disrupted the natural N balance in the environment, resulting in problems such as soil hardening, nutrient imbalance, microbial reductions, and greenhouse gas emissions^1,2^. Therefore, improving soil conditions and the quality of cultivated land were critical issues for sustainable agricultural development.

Maize is a widely cultivated food crop that occupies a substantial portion of agricultural land and contributes appreciably to global production. However, the waste creation and burning of straw after maize harvesting have caused severe environmental problems, such as air and water pollution. In China, the annual production of straw ranges from 600 to 700 million tonnes, and the effective utilisation rate is less than 50%^3,4^. The effective and rational use of waste resources is a measure that must be addressed to promote sustainable development in the fields of agriculture, economy, and environment. The conversion of crop straw to biochar and its application to farmland has several benefits. This method can not only recycle agricultural waste but also significantly improve the soil structure, fertility, and microbial ecosystem, which is conducive to carbon sequestration and C emission reduction in farmland^5–7^.

Biochar typically refers to a carbon-rich product produced from incomplete combustion of biomass, such as agricultural and forestry waste, under low oxygen and high temperature^8^. As a resource recycled from agricultural waste, biochar has attracted wide attention because of its ability to improve soil moisture and nutrient conditions, crop yield, and fertiliser utilisation^9,10^. The excellent porous structure of biomass is well preserved during pyrolysis endowing biochar with a high surface area and exceptional adsorption capacity^11^. Soil bulk density was lower and soil porosity was increased by biochar application^12,13^. The strong adsorption capacity of biochar can effectively improve the water-holding capacity (WHC) of soil^14,15^. Biochar can improve the physical structure of soil by comprehensively balancing its solid, gas, and liquid phases^16,17^. Biochar is mainly composed of C (30–90%), with averaging 64%^7^. Adding biochar to the soil can directly increase its C stock^18,19^. Owing to its stable aromatic structure, biochar has stable physical and chemical properties and can maintain its effect in soil for over a century after application, thus achieving a true “C sequestration”^20^. Due to its unique structural characteristics, biochar can indirectly affect microbial abundance and activity in soil^21,22^. When applied to soil, biochar can provide a good habitat for its microbes^23^. Furthermore, the microporous structure of biochar reduces the competition for survival among microbes, protecting the population of beneficial soil microbes. The strong adsorption capacity allows biochar to adsorb available nutrients from the soil, thus providing C sources, energy, and mineral nutrients that support microbe survival and reproduction^24^.

An excessive amount of biochar can reduce the microbial abundance and enzyme activity in the soil and even lead to yield reductions^25^. According to the principle of microbial C and N balance in soil^26^, there is a fixed ratio between the C and N sources required to maintain normal microbial activity. Excessive sources of C can disrupt this balance and reduce microbial abundance.

Current research focuses mainly on the impact of biochar on a specific aspect of the soil, such as its physical structure, C and N storage, or microbial quantity. However, soil is a complex ecosystem, with multiple properties that interact with each other. Therefore, there is an urgent need to evaluate the changes and potential interrelationships in various soil properties resulting from the addition of biochar systematically.

In this study, we aim to enhance the soil ecological environment through the combination of biochar and nitrogen NF, so that soil quality and spring corn yield can be synergistically increased. Here, we formulated three hypotheses. Hypothesis 1: the application of biochar and NF to the soil can directly regulate its physical structure, C and the N stocks, and C-to-N ratio (CNR). Hypothesis 2: the combined use of biochar and NF can increase the microbial abundance and enzyme activity related to C and N in soil by regulating its C and N balance. Hypothesis 3: the interactive effect of biochar and NF can comprehensively improve soil quality parameters, spring maize yield (SMY), and N-use efficiency (NUE). Improving soil quality and maize yields by improving SME has significant implications for promoting sustainable agriculture in the study area and other similar regions.

## Results

### Changes in the physical properties of the soil

**Figure 1:**
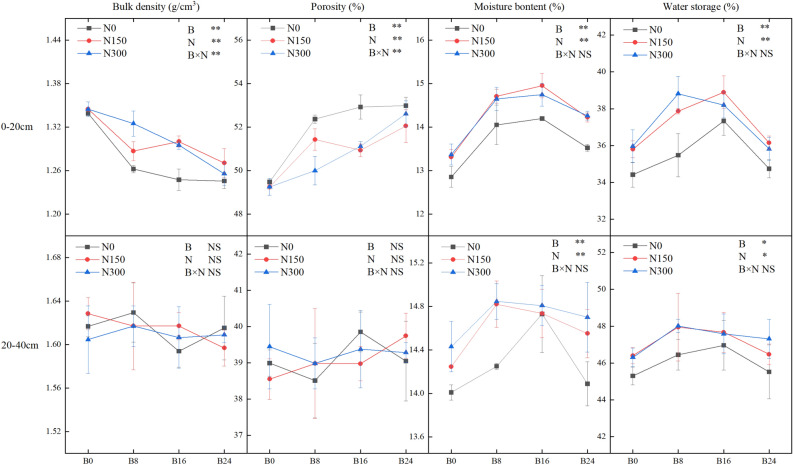
Effects of the combined application of biochar and NF on the physical properties of the soil. Treatments: B0, B8, B16, and B24 corresponding to the application of 0, 8, 16, and 24 t/ha of biochar, respectively. N0, N150, and N300 corresponding to the application of 0, 150, and 300 kg/ha of NF. The error bars indicate SE. **, *and NS indicate an extremely significant difference, a significant difference and no significant difference among treatments (*P* = 0.05, Tukey’s HSD test).

A two-way analysis of variance (ANOVA) using biochar and NF (Fig. 1) reveals the significant impact of both amendments on bulk density, porosity, water content, and WHC of soil within the 0–20 cm layer (*P* < 0.01). Moreover, they exhibit a significant interactive effect on bulk density and porosity. In the 20–40 cm layer, both biochar and NF have a significant impact on water content and the calculatory WHC of the soil (*P* < 0.05).

In the soil layer between 0 and 20 cm, an increase in the level of biochar application resulted in a decrease in bulk density and an increase in porosity, as indicated in Fig. 1. At the N0 level, treatments B8, B16, and B24 resulted in a significant decline in bulk density of 5.80%, 6.91%, and 7.03%, respectively, compared to treatment B0. These reductions were 3.96%, 2.97%, and 5.19% at N150 level and 1.12%, 3.34%, and 6.29% at N300 level (*P* < 0.05). No significant differences were observed between biochar treatments at each NF application level in the soil layer of 20–40 cm. In both soil layers, the application of biochar alone resulted in a greater decrease in bulk density and a greater increase in porosity compared to treatments N150 and N300. Therefore, it can be inferred that the application of biochar alone contributed to a lower bulk density, whereas the addition of the application of NF helped to reduce the impact of high-level biochar on soil compactness.

Figure 1 illustrates that the addition of biochar (B8, B16, and B24) in the 0–40 cm soil depth significantly augmented water content by 0.57–10.39% and 1.88–10.53%, besides WHC by 0.5–8.46% and 0.97–8.63%, with and without NF treatment (N0), respectively. Moreover, at each biochar application level, N150 and N300 treatments substantially increased the water content by 0.16–8.63% and WHC by 2.16–7.91%, relative to the N0 treatment. The findings indicate that incorporating biochar and NF into the soil can enhance its WHC, with further benefits observed from their combined application.

**Figure 2:**
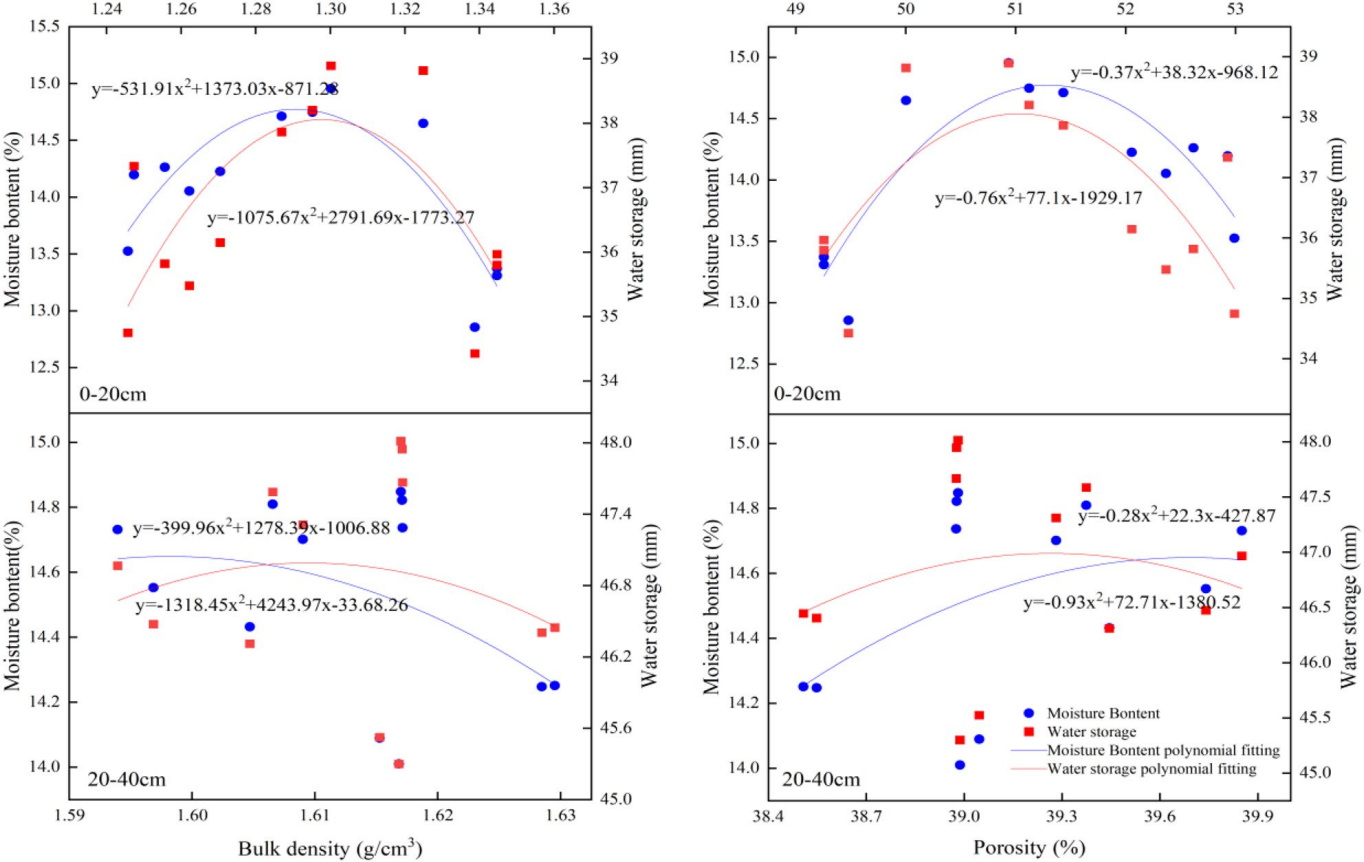
Relationship between the bulk density, porosity, and WHC of the soil.

The influence of biochar, NF, and their interplay on the soil's bulk density and porosity proved substantial solely within the 0–20 cm layer. Hence, solely an analysis was conducted on the correlation between bulk density, porosity, and WHC of the soil in this depth. Figure 2 illustrates an unimodal association of the WHC with both bulk density and porosity. The analysis of the quadratic equation fit found that the highest levels of water content and WHC in the soil layer of 0–20 cm correlated to bulk density values of 1.29 g/cm^3^ and 1.30 g/cm^3^ and porosity values of 51.78% and 50.72%. Results suggest loose or compact structures can reduce soil's WHC.

**Figure 3:**
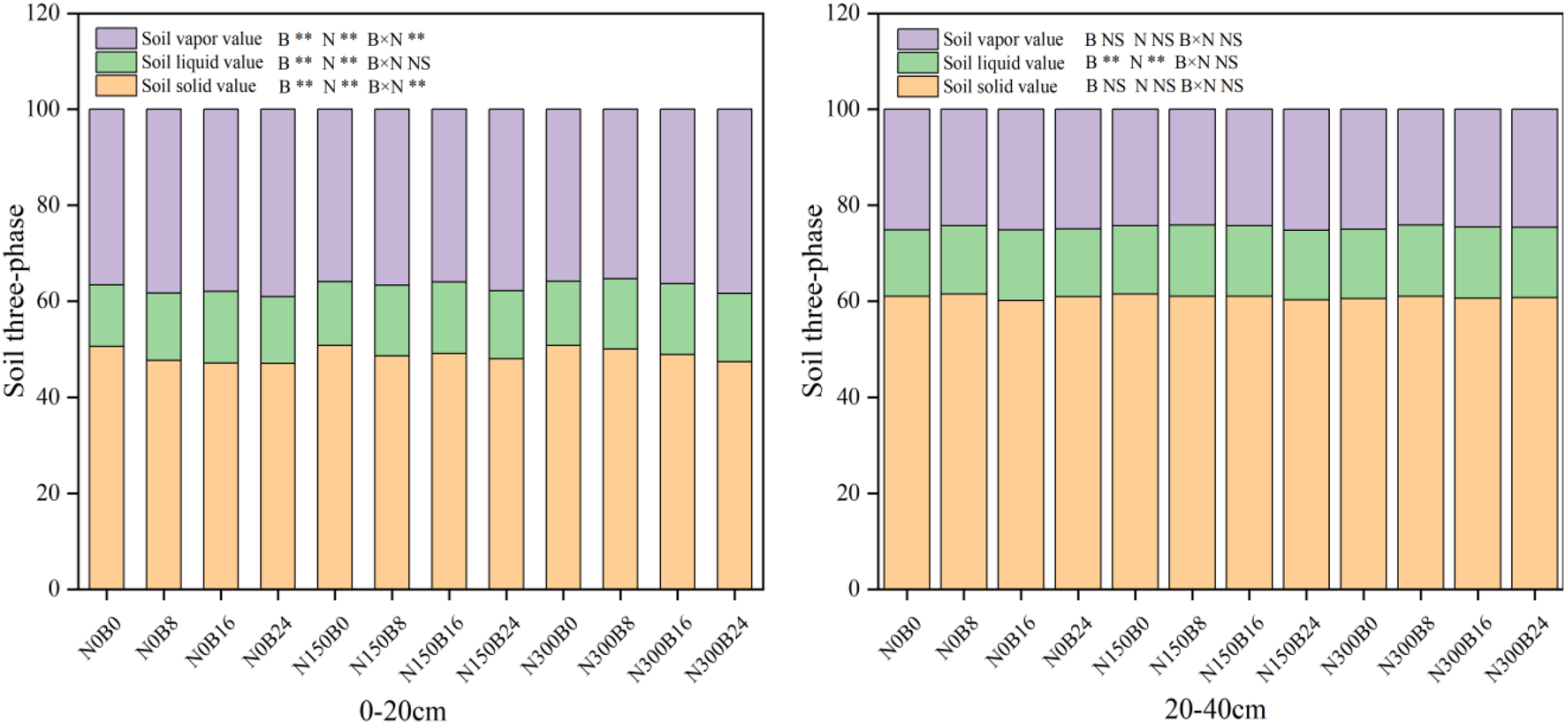
Effects of combined application of biochar and NF on the solid, liquid, and gas phases of the soil. Treatments: B0, B8, B16, and B24 corresponding to the application of 0, 8, 16, and 24 t/ha of biochar, respectively. N0, N150, and N300 corresponding to the application of 0, 150, and 300 kg/ha of NF. **, *and NS indicate an extremely significant difference, a significant difference and no significant difference among treatments (*P* = 0.05, Tukey’s HSD test).

Figure 3 illustrates that biochar and NF significantly influenced the content of the solid, liquid, and gas phases of the soil in the 0–20 cm layer. There was also an observable interactive effect on the gas and solid-phase content. In the 20–40 cm soil layer, biochar and NF had a significant impact on the content of the liquid phase. The application of biochar and NF had a significant impact on the soil structure by altering the composition of its upper 20 cm.

In the 0–20 cm layer of soil, the solid-phase content significantly decreased as the biochar application level increased. Treatment B8 in particular reduced solid-phase content by 5.72%, 6.83%, and 6.95% at levels N0, N150, and N300, respectively, compared to treatment B0. The values were 4.30%, 3.32%, and 5.53% lower under treatment B16 and 1.48%, 3.69%, and 6.63% under treatment B24, respectively. At every biochar application level, applying NF significantly increased the solid-phase content of the soil. Nonetheless, there was no significant distinction observed between the two levels of NF application. The liquid-phase content pertains to the soil's water content examined in the preceding section. Introducing biochar significantly inflated the soil's gas-phase content by 1.38–6.90% at each NF application level within the 0–20 cm soil layer. By comparison, NF significantly reduced the gas-phase content of the soil, with no significant differences between the two NF application rates. The use of biochar and NF only displayed considerable alterations in the soil's liquid-phase content, in the layer ranging from 20 to 40 cm.

**Figure 4:**
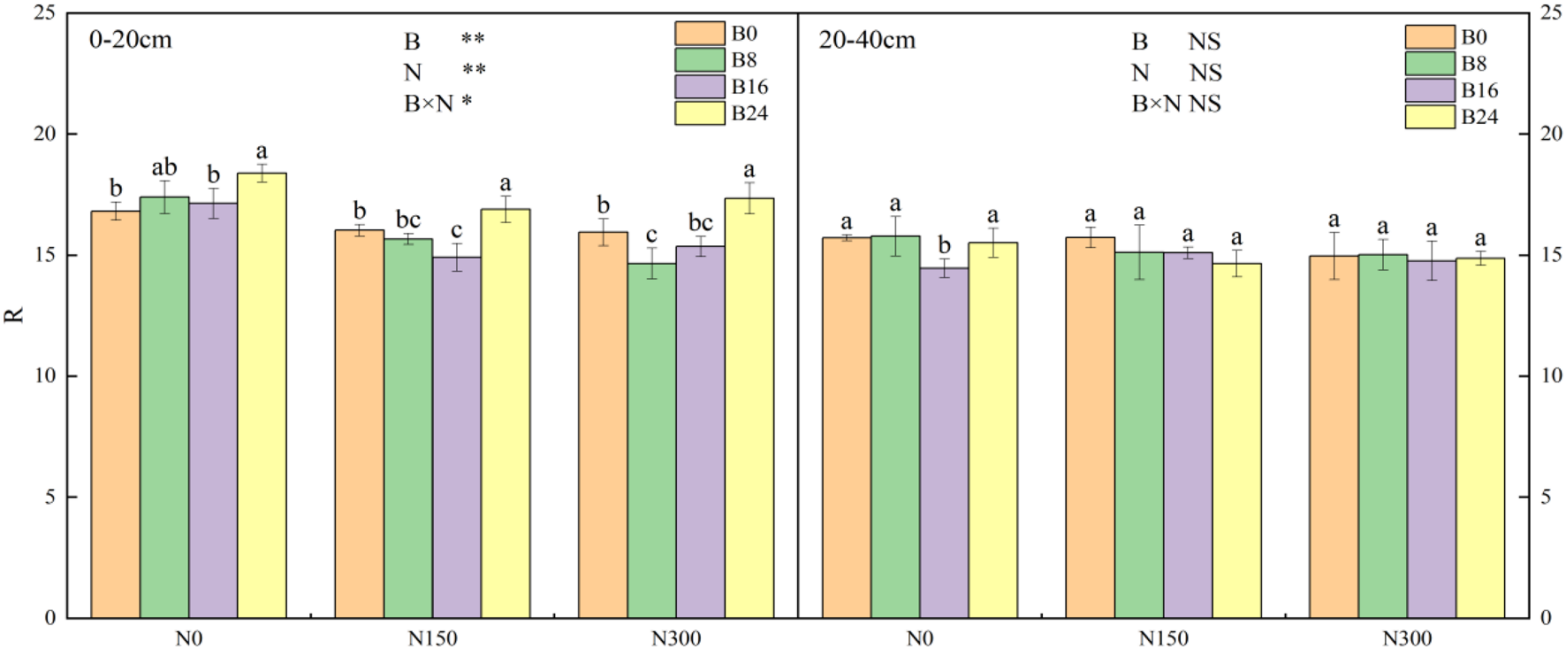
Effects of the combined application of biochar and NF on R. Treatments: B0, B8, B16, and B24 corresponding to the application of 0, 8, 16, and 24 t/ha of biochar, respectively. N0, N150, and N300 corresponding to the application of 0, 150, and 300 kg/ha of NF. **, *and NS indicate an extremely significant difference, a significant difference and no significant difference among treatments (*P* = 0.05, Tukey’s HSD test).

Figure 4 demonstrates that both biochar and NF had a significant impact on deviating from the ideal ratio in actual soil (R) in the 0–20 cm soil layer, with a notable interactive effect between them. When compared to treatment B0, treatments B8, and B16 considerably reduced R by 2.24% and 6.96% at level N150 and by 13.23% and 9.08% at level N300, whereas treatment B24 increased R by 5.39% and 2.68% at levels N150 and N300, respectively. Treatments N150 and N300 led to significant reductions in R. At level B8, R decreased by 9.94% and 15.77%, respectively. At level B16, reductions of 12.97% and 10.39% were observed, and at level B24, 8.12% and 5.69% reductions were observed, respectively. In the 0–20 cm layer of soil, the application of biochar increased R. However, introducing a moderate amount of NF, in addition, reduced R, bringing the experimental soil closer to the ideal solid–liquid–gas-phase ratio (SLGR).

### Organic carbon (OC) and TN content and CNR of the soil

**Figure 5:**
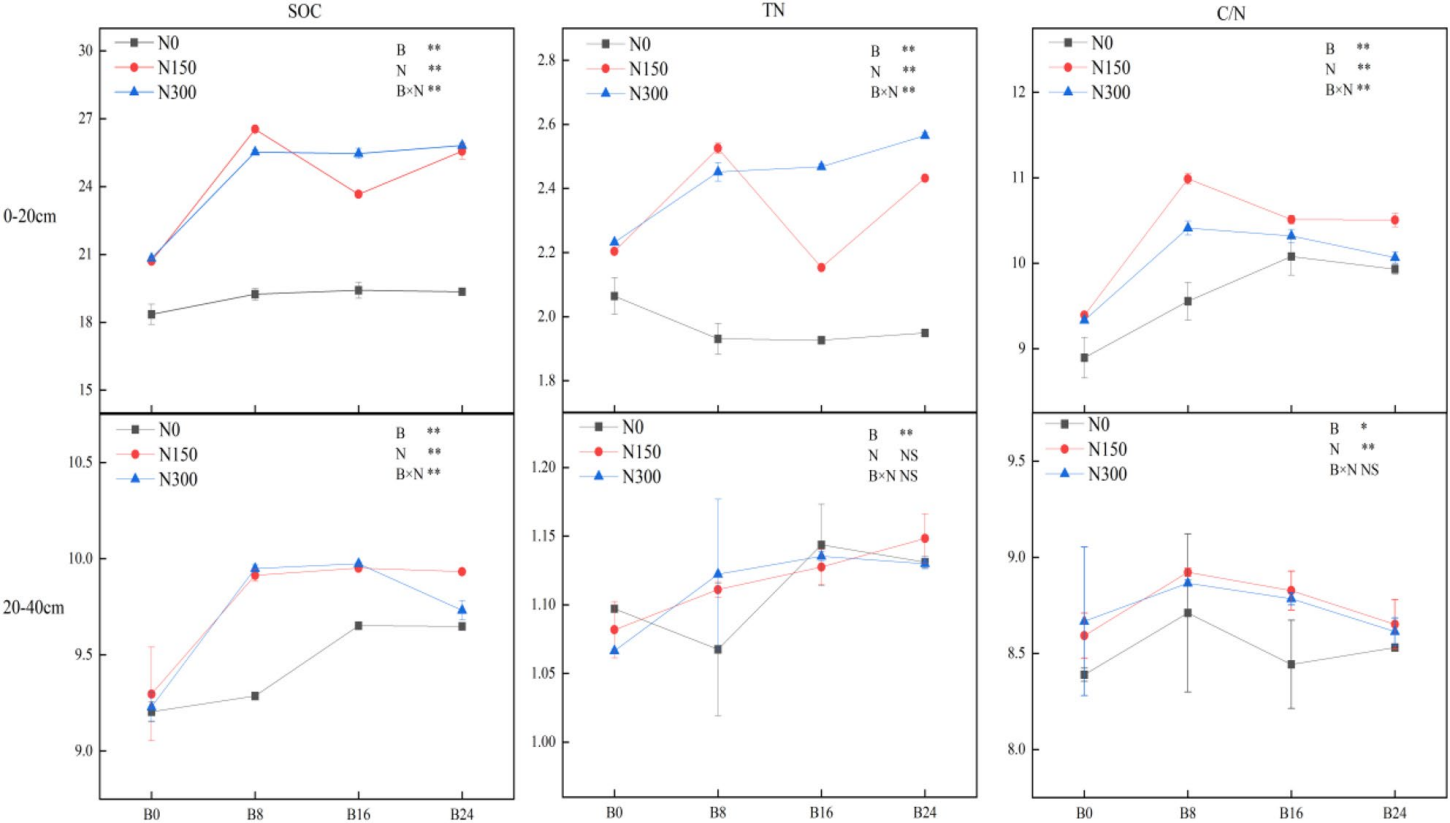
Effects of combined application of biochar and NF on the OC and TN content and CNR of the soil. The error bars indicate SE. Treatments: B0, B8, B16, and B24 corresponding to the application of 0, 8, 16, and 24 t/ha of biochar, respectively. N0, N150, and N300 corresponding to the application of 0, 150, and 300 kg/ha of NF. **, *and NS indicate an extremely significant difference, a significant difference and no significant difference among treatments (*P* = 0.05, Tukey’s HSD test).

Figure 5 illustrates that both biochar and NF had a significant impact on the OC content and CNR of the soil in both layers, exhibiting a notable interactive effect. The OC content and CNR increased as the biochar application level increased at each of the N0, N150, and N300 levels in the 0–20 cm and 20–40 cm soil layers, reaching their highest values at level B24. Moreover, the addition of NF led to a more significant increase than biochar alone. The TN content in both soil layers experienced a significant increase of 4.95–16.76% and 5.01–26.55%, correspondingly, upon application of biochar solely (N0) or together with NF (N150 and N300). The increase was more pronounced with both amendments. There was no noteworthy difference between the NF treatments at the matching biochar application level. The application of biochar alone and along with NF both significantly upraised the CNR of the soil in the 0–20 cm layer. Only soil treated with the combined B16 and N150 and B24 and N150 treatments showed increased CNR in the 20–40 cm layer. Furthermore, the application of other treatments resulted in a significant decrease in the soil's CNR.

### Microbial biomass (MB) of the soil

**Figure 6:**
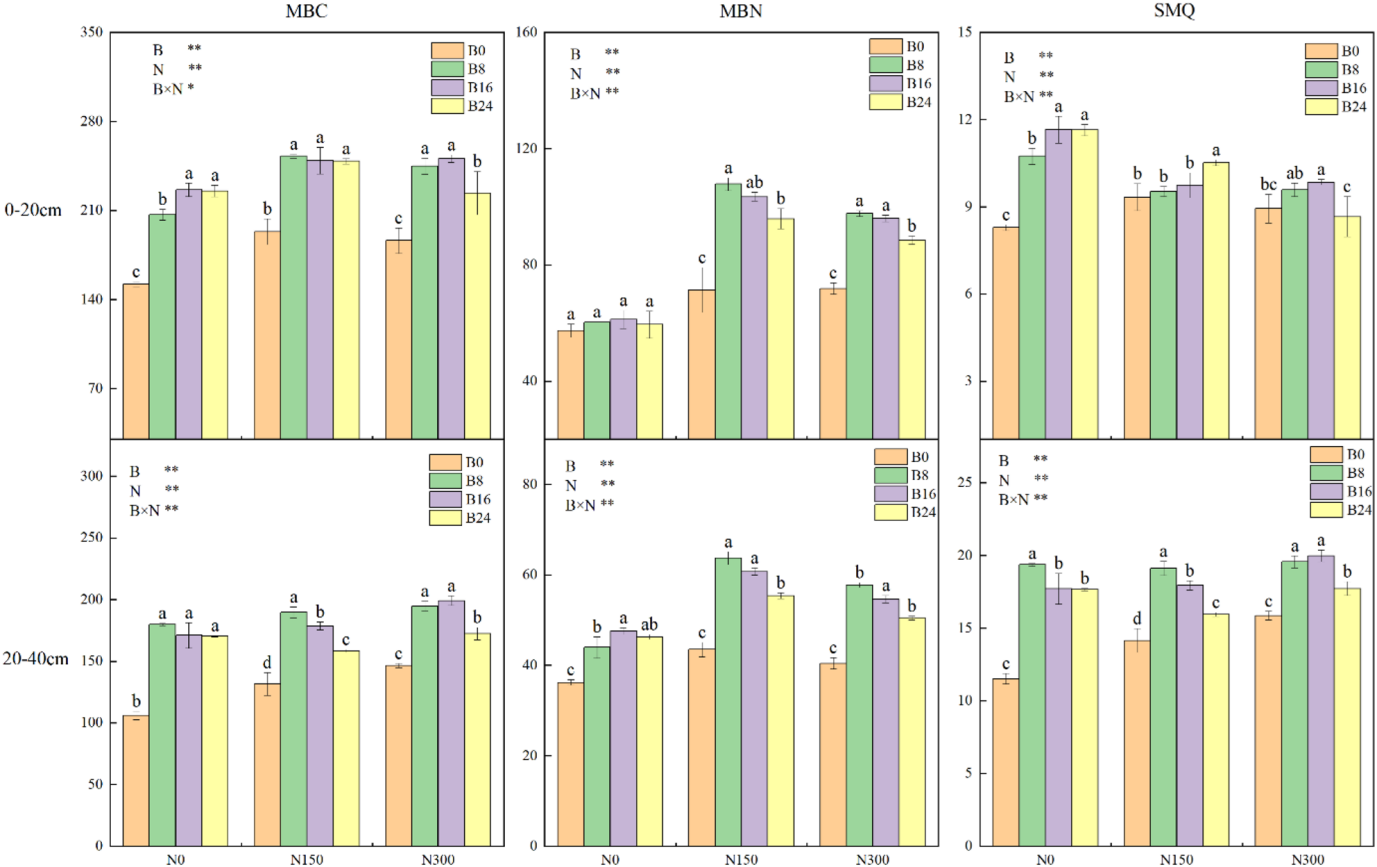
Effects of combined application of biochar and NF on the content of MBC and MBN, as well as the MQ, of the soil. The bars and error bars indicate the mean + SE. Treatments: B0, B8, B16, and B24 corresponding to the application of 0, 8, 16, and 24 t/ha of biochar, respectively. N0, N150, and N300 corresponding to the application of 0, 150, and 300 kg/ha of NF. **, *and NS indicate an extremely significant difference, a significant difference and no significant difference among treatments (*P* = 0.05, Tukey’s HSD test).

Figure 6 depicts the significant impact of both biochar and NF on the MBC and MBN content, as well as the microbial quotient (MQ) of the soil in both layers. However, there was a significant interactive effect. In the 0–20 and 20–40 cm soil layers, while holding the NF application level constant, the MBC and MBN content both witnessed an increase and a subsequent decrease as the biochar application level increased. It is pertinent to mention that both the soil properties are significantly higher in the 0–20 cm layer when compared to the 20–40 cm layer in all treatments (Fig. 6). Treatment B8 produced the highest MBC content at levels N150 and N300 in each soil layer, with values of 252.78 and 199.13 g/kg, respectively. The combination of biochar and NF resulted in a greater MBC content than biochar alone. Both treatments N150 and N300 resulted in a significant increase in MBC content compared to treatment N0 at each biochar application level. However, there was no significant difference between the two NF application levels. The addition of biochar resulted in an increase in both soil layers' MBN content at every NF application level. The MBN content was greatest under treatment N150 and then under treatments N300 and N0 for each biochar application level. The soil's MQ is determined by its MBC content to OC content ratio, providing valuable insight into its C pool activity. Only treatments B8 and B16 significantly increased MQ by 4.45–30.95% and 20.01–68.06%, respectively, compared to treatment B0 in both soil layers, regardless of the level of NF application.

### Soil enzyme activities

**Figure 7:**
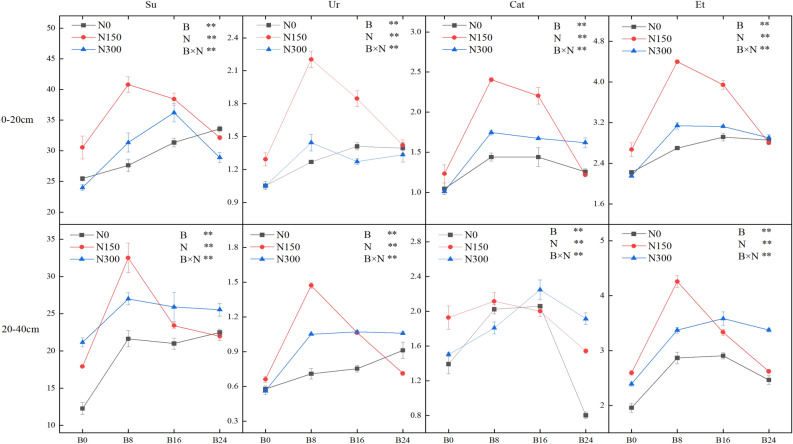
Effects of combined application of biochar and NF on soil enzyme activities. The error bars indicate SE. Treatments: B0, B8, B16, and B24 corresponding to the application of 0, 8, 16, and 24 t/ha of biochar, respectively. N0, N150, and N300 corresponding to the application of 0, 150, and 300 kg/ha of NF. **, *and NS indicate an extremely significant difference, a significant difference and no significant difference among treatments (*P* = 0.05, Tukey’s HSD test).

Both biochar and NF had a significant impact on soil enzyme activities in both soil layers, as evidenced by their notable interaction. Furthermore, it is worth noting that technical term abbreviations have been explained upon first use. The activities of sucrase (Su), urease (Ur), and catalase (Cat) in the 0–20 and 20–40 cm soil layers increased considerably with the application of both amendments, and their combined use resulted in an even greater increase (Fig. 7). In the layers of soil from 0–20 to 20–40 cm, the optimal Su activity was discovered through the combined implementation of B8 and N150 treatments, displaying values of 40.80 and 32.49 mg g^−1^ d^−1^, correspondingly. With treatments of N150 and N300, the usage of biochar notably increased Ur activity in both soil layers by 10.14–122.98%. At every degree of biochar application, Ur activity elevated initially and subsequently shortened as the NF application intensified. The activity of Ur was significantly greater under the N150 treatment when compared to treatments N300 and N0. In both layers of soil, the highest level of Cat activity was found when the B8N150 and B16N300 treatments were combined, at values of 2.40 and 2.11 mg g^−1^ d^−1^, respectively.

The enzyme activity parameter, Et, was used to characterise the enzyme activity of the experimental soil samples. By transforming the measured values into relative ones, a thorough assessment was conducted on the alteration pattern of enzyme activity regarding diverse measurement units. The outcome revealed a steady increment in Et as the biochar application level rose, followed by a decrease (Fig. 7) at the equivalent NF application level. In the soil layers of 0–20 and 20–40 cm, the most significant Et values were observed in B8 and N150 combinations, with respective measures of 4.40 and 4.26 mg·g^−1^·d^−1^. The results indicated a 98.00% and 117.63% increase compared to the combined application of treatments B0 and N0.

### Correlations among the physical structure, CNR, and biological properties of the soil

**Figure 8:**
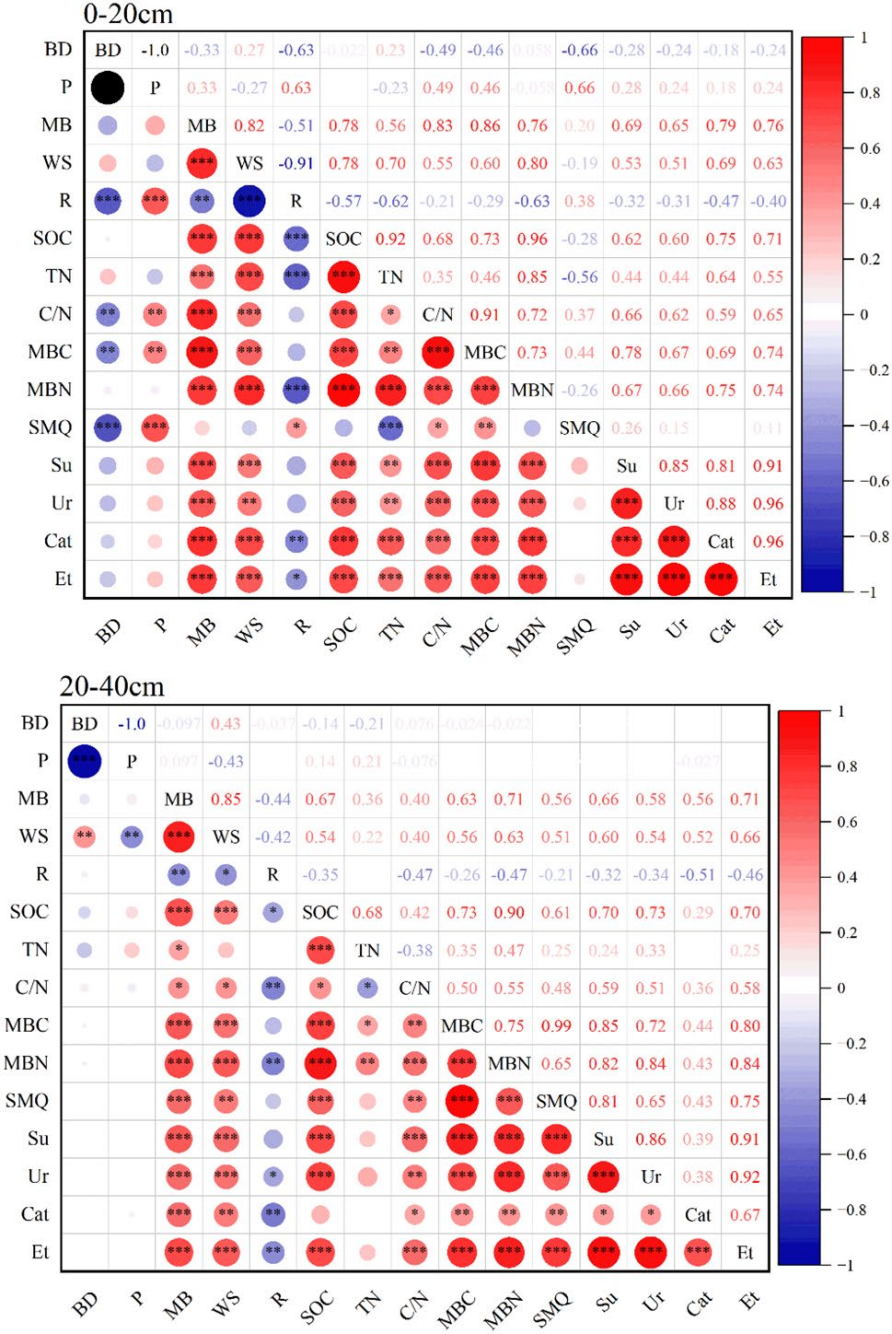
Correlation between SMY and soil properties. The figure is divided into two diagonals. The lower triangle on the left is the correlation of ptwo indicators, *indicates a significant difference at the *P* < 0.05 level, **indicates a significant difference at the *P* < 0.01 level, *** indicates a significant difference at the *P* < 0.001 level, red represents a positive correlation, blue represents a negative correlation, the darker the colour, the greater the correlation. The figures in the upper right triangle are pairwise indicator correlation coefficients.

The R value of the soil can indicate its structural quality. A lower R value suggests that the soil is nearer to the ideal state, which indicates better soil structure. Figure 8 indicates that in the 0–20 cm layer, the soil's R value had a marked and negative correlation with its WHC (MB and WS) as well as its C and N content (OC and TN content). Furthermore, a significant negative correlation was observed between the soil's MBN content and Et values. These findings suggest that a decrease in the R value results in improved C and N content and overall microbial properties of the soil. The OC/TN ratio is considered an indicator of soil CNR. The soil's CNR displayed extremely significant and positive correlations with WHC, MBC, MBN, and enzyme activities (Su, Ur, Cat, and Et) in the 0–20 cm layer. The R value exhibited similar relationships with various soil properties in both the 20–40 cm layer and the 0–20 cm layer. Furthermore, the CNR was significantly positively correlated with the soil's WHC and OC content, and it was extremely significantly positively correlated with its MB content, MQ, various enzyme activities and overall enzyme activity.

### Relationship between SMY and soil properties

**Figure 9:**
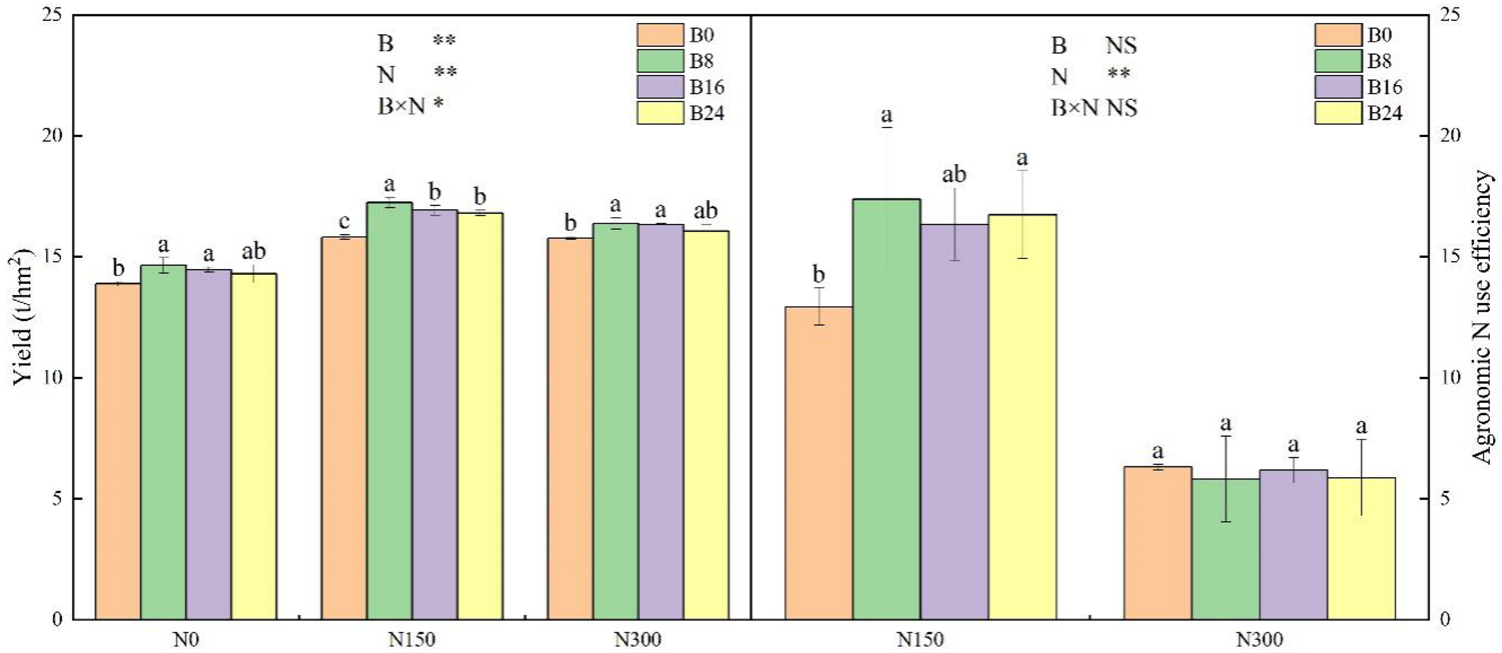
Effects of the combined application of biochar and NF on SMY and NUE. The bars and error bars indicate the mean + SE. Treatments: B0, B8, B16, and B24 corresponding to the application of 0, 8, 16, and 24 t/ha of biochar, respectively. N0, N150, and N300 corresponding to the application of 0, 150, and 300 kg/ha of NF. **, *and NS indicate an extremely significant difference, a significant difference and no significant difference among treatments (*P* = 0.05, Tukey’s HSD test).

The use of either biochar or NF individually increased SMY. However, the increase was more substantial when both were applied simultaneously, as shown in Fig. 9. When treatments B0 and N0 were applied, there was no fertiliser present, and when NF was used alone (treatments B0 and N150), the SMY was significantly lower than when both B8 and N150 were used together, achieving the highest SMY. The combination of treatments B8 and N150 produced a yield that was 24.25% and 9.04% higher than the combination of treatments B0 and N0 and treatments B0 and N150, respectively. At every biochar application level, the agronomic efficiency of N (NAE) was significantly higher in the N150 treatment compared to the N300 treatment (Fig. 9).

Correlation analysis of SMY and soil properties in both layers (Table 1) indicates significant positive correlations between SMY and WHC (water content and water storage capacity), OC content, TN content (in the 0–20 cm soil layer), CNR, MB content, and soil enzyme activities in both layers. However, SMY showed a significant negative correlation with the R value of the soil.

**Table 1:** Correlation between SMY and soil properties.

Soil property metric	BD	P	MB	WS	R	SOC	TN	C/N	MBC	MBN	SMQ	Su	Ur	Cat	Et
SMY and soil properties in the 0–20 cm layer	0.15	− 0.15	0.65***	0.75***	− 0.63***	0.89***	0.80***	0.65***	0.66***	0.89***	− 0.26	0.58***	0.60***	0.64***	0.64***
SMY and soil properties in the 20–40 cm layer	0.06	0.06	0.62***	0.53***	− 0.63***	0.66***	0.21	0.56***	0.47**	0.79***	0.39*	0.68***	0.60***	0.46**	0.69***

## Discussion

This study showed that the combination of biochar and NF has a synergistic positive effect on soil structure and C and N stocks. Optimising the SLGR, this approach improves the soil structure and improves the soil’s ability to retain water and nutrients, effectively preserving newly added C and N in the plough layer soil^27^. The application of biochar alone resulted in a deviation of the soil SLGR in the 0–20 cm layer from the ideal value, whereas the inclusion of NF reduced the R value, bringing the experimental soil closer to the optimal state. Biochar has a stable structure that can remain unchanged in the soil for several decades or even over a century^28^. The long-term fixed-point experiment conducted in this study demonstrated that the application of biochar only during seeding in the first year still led to an improvement in soil structure after five years, indicating the sustained effect of biochar after being returned to the field. In addition, the combined application of biochar and NF provides soil with direct sources of C and N, effectively increasing its C and N stocks and CNR. Biochar produced from the burning of maize straw can contain over 70% C^29^ and possesses a stable aromatic hydrocarbon structure, making it resistant to chemical changes in the short-term, playing a role in C sequestration^30^. The biochar used in this study contained 72.12% C. Here, even after five years, the application of biochar, alone or combined with NF, caused an increase in soil OC content of the soil in the layer of 0–20 cm, with a greater increase observed when applied with NF. Furthermore, biochar has a remarkable adsorption capacity that enables it to adsorb large amounts of nitrate N and ammonium ions, increasing the TN content of the soil and a decrease in its N losses^31,32^. This study showed that the application of biochar with NF significantly increased the CNR of the soil in the 0–20 cm layer, with biochar playing a dominant role. Biochar is rich in carbon and increases soil organic carbon content when applied. The adsorption capacity of biochar for N is lower than its ability to increase the OC content, resulting in a greater increase in C content than N content and an increase in the CNR. In addition, although the biochar itself does not move in space, the conventional land preparation method before sowing is shallow rotary tillage. After five years, biochar will be incorporated into the deeper soil.

**Figure 10:**
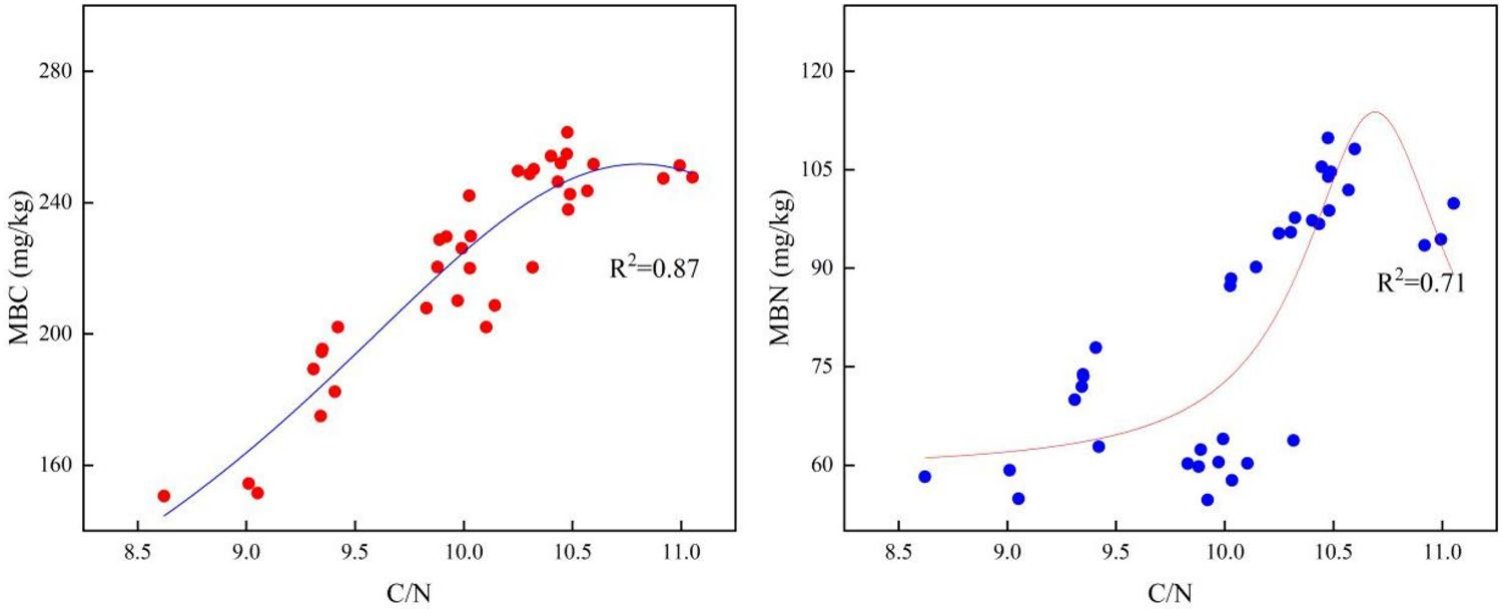
Relationships between the MBC and MBN content of the soil and its CNR.

In recent years, most research has focused on the effects of the sole application of biochar on MB and enzyme activity in soil, with scant attention given to the interactive effect of biochar and NF on the biological properties of soil. In this study, the combination of biochar with NF increased MBC and MBN content, MQ, Su, and Ur activity, and Et compared to biochar alone. Correlation analysis of the CNR of the soil with MB and enzyme activity (Fig. 8) revealed a positive relationship between the CNR and the microbial properties of the soil. Figure 10 shows that the application of biochar alone resulted in an excessively high CNR, leading to a decrease in MBC and MBN content. Research^33^ has shown that 20–25 parts of C and one part of N are required to maintain normal microbial activity. When there is an excess of C sources and a relative lack of N sources, the inherent C and N imbalance in the soil will force microbes to form cell material based on the available N. This results in a lower microbial population that cannot reach its maximum potential, leading to a reduced ability to decompose organic matter (OM). Under these conditions, the addition of inorganic N to supplement the N deficiency in the soil can increase the microbial population. Based on this reasoning, if biochar is applied alone, excessive C sources without sufficient N will fail to meet the needs of microbial activity, suppressing the microbial population and activity. This study showed that the addition of 150 kg/ha of NF significantly increased the content of MBC and MBN compared to the biochar-only treatment. This indicates that the added N sources re-established a suitable CNR for microbial activity, whereas the increase in the amount of C and N sources improved microbial abundance. The combination of biochar and NF indirectly increased MB and enzyme activity in the soil by directly affecting its CNR. Therefore, the effects of biochar and NF on the physical structure, C and N stocks, and biological properties of the soil (Fig. 8) suggest that their combined use may improve the structure of the soil and provide a favourable living environment for microbes, thus improving the abundance and enzyme activity. Furthermore, the increased C and N stocks resulting from their combined application provided C and N sources for microbial growth and reproduction, whereas a reasonable CNR further improved the biological properties of the soil.

Current research consensus suggests that the use of biochar promotes crop growth and yields, and that combining biochar and NF produces a positive effect on crop production^34^. However, the optimal application rate of biochar varies depending on the preparation process and soil properties at the experimental site^35,36^. In this study, both biochar and NF significantly improved the grain yield of spring maize, with the highest yield achieved with the application of 8 t/ha of biochar and 150 kg/ha of NF. From a biochar application perspective, a slight decrease in yield was observed in treatment B24, but it remained higher compared to biochar-free treatments. The reduction in crop yield with the application of high-level biochar may be ascribed to the high C content of the biochar, which led to a high CNR, which reduced the availability of N in the soil. Regarding the application of NF, the yield increased with an increase in the level of NF application, but there was no significant difference between treatments N150 and N300. NAE analysis revealed a significantly higher NUE under treatment N150 than under treatment N300. Excessive application of NF resulted in increased yield but significantly decreased NUE, leading to fertiliser waste and surplus N surplus in the soil. These results indicate that the combined application of moderate amounts of biochar (8 and 16 t/ha) and NF (150 kg/ha) can synergistically increase crop yield and NUE. Considering economic benefits, the application of 8 t/ha of biochar is more profitable.

The correlation analysis between yield and bulk density and porosity in each soil layer revealed that excess bulk density and porosity were detrimental to crop growth. When biochar was applied alone, the bulk density was less than 1.26 g/cm^3^, resulting in loose soil, difficult to till. High yields were obtained when treatments B8 and B16 were combined with NF, with corresponding bulk densities ranging from 1.29 to 1.30 g/cm^3^. This indicates that only a suitable soil structure can significantly increase crop yield (Table 1). The increase in yield due to the combined use of biochar and NF may be attributed to their ability to bring the soil SLGR to the ideal value, which enhanced the ability of the soil to retain water and nutrients.

The correlation analysis between the yield and the physical properties of the soil in the two layers (Table 1) revealed that the improvement in the crop yield observed with the combined use of biochar and NF can be attributed to their ability to improve the physical structure of the soil, regulate its CNR, and boost its MB and enzyme activity. Moderate levels of biochar (B8 and B16) and NF (N150) resulted in the highest yield, NUE, microbial abundance, MQ, and enzyme activity related to the cycle of C and N cycling in the soil. Excessive application of NF (N300) reduced microbial abundance and enzyme activity in the topsoil. One reason treatment N300 did not significantly increase yield compared to treatment N150. Although the highest soil C and N stocks were achieved with the highest-level application of biochar and NF, the optimal overall effect on biological properties, yield, and NUE was achieved with the moderate application of biochar and NF. This approach improved soil structure, regulated the balance of C and N in the soil, and improved the biological and chemical properties of the soil, leading to a synergistic increase in both yield and NUE.

Other studies have shown that biochar has a certain sustained effect on crop yields. Major et al*.*^5^ reported that the application of 20 t/ha of biochar did not affect the yield in the first year of a multiyear maize and soybean rotation experiment. However, in the following three years, the yield gradually improved and eventually increased by 140% in the fourth year. Yuan et al*.*^37^ observed that the combined application of biochar and NF increased crop yield. The best result was achieved with 10 t/ha of biochar and 300 kg/ha of NF, which increased the yield by 26.9% compared to the control. This study also confirmed that biochar had a significant positive effect on soil quality and yield, even after five years of application, with a pronounced sustained effect.

In conclusion, the combined use of biochar and NF significantly reduced the deviation from the ideal soil in terms of SLGR, leading to a physical structure that more closely resembled the ideal soil. The application of biochar alone and in combination with NF appreciably increased the OC and TN content of the soil, as well as its CNR. Moderate application of biochar (8 and 16 t/ha) combined with NF (150 kg/ha) resulted in the highest MB, MQ, and enzyme activity related to C and N cycling in the soil by adjusting the suitable CNR of the soil to the value for microbial activity. The application of 8 t/ha of biochar and 150 kg/ha of NF was the most effective in improving soil quality, SMY, and NUE. This study provides a valuable reference for improving soil conditions, SMY, and NUE in regions similar to the golden maize belt. However, more research is necessary to investigate the mechanisms of biochar and NF in improving soil and crop yields under varying climatic and soil conditions.

## Methods

### Overview of the experimental site

**Figure 11:**
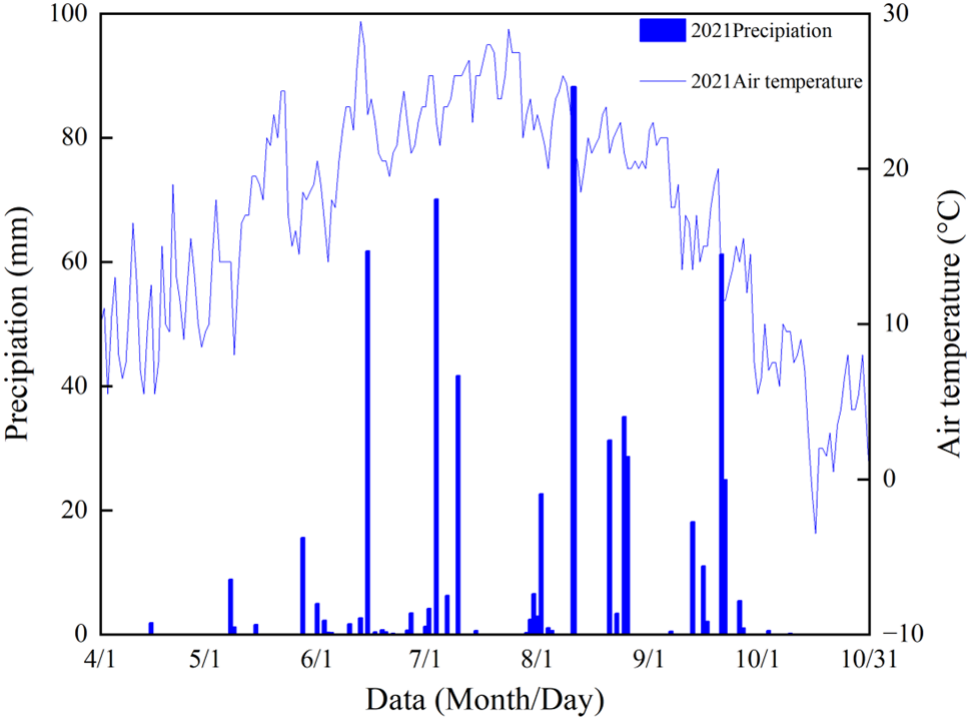
Precipitation during the maize-growing season in the study area (mm).

This study was conducted in the western Liaohe Plain of China (43°86′N, 121°50′E), a region located in the golden maize belt (near 45°N). The soil type in this area is sandy loam. Specifically, in the Fanjiayao Village, Xiaojieji Township, Kailu County, Tongliao City, located in Inner Mongolia, a region with a temperate continental monsoon climate characterised by four distinct seasons and a rainy season that coincides with hot weather. The annual precipitation in the area is concentrated in summer and averages 266.6 mm. The average temperature is 18.3 °C, and the annual sunshine duration is 1,817.8 h (Fig. 11). Spring maize is the main crop in the region.

Before spring planting in 2017, baseline nutrient values were determined for the soil layer at depths of 0–40 cm. The soil had an OM content of 18.05 g·kg^−1^, a total N (TN) content of 0.41 g·kg^−1^, an available N content of 55.12 mg·kg^−1^, available phosphorus (P) content of 12.64 mg·kg^−1^, an available potassium (K) content of 177.61 mg·kg^−1^, a pH of 7.2, a bulk density of 1.4 g/cm^3^, and a water content of 20.11%.

### Experimental materials

The biochar used in the experiment was obtained from Shenyang Longtai Bioengineering Co., Ltd. and was produced by pyrolysing maize straw under low oxygen. The biochar is burned at 400 °C for 2–3 h. It had a pH of 9.88 and contained 72.12% C, 1.21% N, 0.68% P, and 1.21% K.

### Experimental design

A split-plot design was employed for the experiment. The main plots received four biochar treatments (B0, B8, B16, and B24), corresponding to the application of 0, 8, 16, and 24 t/ha of biochar, respectively. Subplots were subjected to three NF treatments (N0, N150, and N300), involving the application of 0, 150, and 300 kg/ha of NF urea with a pure nitrogen content of 46%, respectively. These treatments were implemented during the tasselling stage. The experiment was replicated three times, resulting in 36 subplots. Encompassing an area of 27 m^2^, each subplot had nine 5 m-long rows spaced 60 cm apart. The Jingke 968 variety was used for the experiment, with a planting density of 75,000 plants/ha. The plots were irrigated four times during the growth period. Each time, each plot received 750 m^3^/ha of water. All other management practices were consistent with those used in conventional field production.

In spring 2017, the biochar was dispersed over the soil surface before sowing and then mixed into the plough layer to a depth of approximately 15 cm using rotary tillage. Five years later, in the fall of 2021, soil samples were collected from the topsoil layer at depths of 0–40 cm for testing.

## Sampling and metric measurement methods

### Sampling

After the spring maize was harvested, five evenly distributed sampling points were selected within each subplot. Using a soil core sampler with a standard volume of 100 cm^3^, undisturbed soil samples were collected from each point at depths of 0–20 and 20–40 cm along the vertical direction of the field. The samples were transported to the lab for soil analysis. Furthermore, at the same sampling points, 200 g of fresh soil was collected and placed in a 4 °C insulated box to determine its C and N content, as well as other metrics related to its biological properties.

During the physiological maturity stage of spring maize in 2021, two rows of evenly grown plants without missing seedlings and broken ridges were harvested from each subplot in the field. The ears were dried naturally, and the best seeds were preserved. Grains in each ear were counted and then all the grains were threshed to determine the thousand-grain weight. Finally, the grain yield was calculated based on these measurements.

#### Measurement metrics and methods

*Physical structure metrics* The soil core sampler and the fresh soil sample were first weighed and then dried in an oven at 105–110 °C for 16 h. After being cooled, they were weighed again to determine the dry weight of the soil (i.e., soil core sampler volume).

$$Soil bulk density (g/{cm}^{3}) = Dry soil weight (g) / soil volume ({cm}^{3})$$

Soil porosity (%) = (Weight of the soil-filled core sampler after approximately 2 h of water absorption—Weight of the core sampler—Weight of the dry soil inside the core sampler)/Volume of the core sampler (cm^3^) × 100%

$$ Soil water content \left( \% \right) = \frac{{\left( {Fresh soil weight {-} Dry soil weight} \right) \left( g \right)}}{Dry soil weight \left( g \right)} \times 100\% $$

$$Soil WHC (mm) = Soil layer depth (cm) \times Soil bulk density (g/{cm}^{3}) \times Soil water content (\%) \times \frac{10 }{1000}$$

$$ Solid phase of soil \left( \% \right) = 1 {-} Porosity $$

$$ Liquid phase of the soil \left( \% \right) = Water content $$

$$ Gas phase of soil \left( \% \right) = Porosity {-} Water content \left( \% \right) $$

In agricultural soils, the ideal solid–liquid–gas-phase ratio (SLGR) of the soil is 50:25:25. A deviation from this ideal ratio in actual soil is defined as ${\text{R}} = \sqrt[2]{{\left( {X - 50} \right)^{2}  + \left( {Y - 25} \right)^{2}  + \left( {Z - 25} \right)^{2} }}$ in the experiment^38^, where X, Y, and Z are the measured values of the solid, liquid, and gas content of the soil, respectively.

*C and N content and biological property metrics of soil* The C and N content of the soil was analysed following the methods described in “Soil Agrochemical Analysis” by Bao^39^. The organic C (OC) content of the soil was determined using the K_2_Br_2_O_7_ oxidation–external heating method, whereas its TN content was measured using an automatic Kjeldahl N analyser. The MB, C (MBC), and N (MBN) content were determined using the chloroform fumigation-K_2_SO_4_ extraction method^40^. The activities of sucrase (Su), urease (Ur), and catalase (Cat) were measured using the 3,5-dinitrosalicylic acid colorimetric method, indophenol blue colorimetric method, and potassium permanganate titration, respectively^41^.

The average enzymatic activity of all the experimental soil samples of the same layer was a reference for calculating the relative enzymatic activity values of the soil samples. The values were subsequently summed to obtain the enzymatic activity parameter $Et=\sum Xi/X$ (Xi and X are the measured and average values of the i^th^ enzymatic activity of the experimental soil sample, respectively)^42^.

### Data treatment and analysis

The data were subjected to a two-way ANOVA using IBM SPSS Statistics 19.0 software (IBM Corp., Armonk, NY, USA). SPSS bivariate correlation analysis was performed to investigate the correlations between the physical and chemical properties of the soil. Multiple comparisons were conducted using the least significant difference method, with significant and extremely significant levels set at *P* < 0.05 and *P* < 0.01, respectively. Microsoft Office 2016 (Microsoft, Seattle, WA, USA) was used to generate tables and compute data, whereas Origin 2022b (Origin Lab, Northampton, USA) was used to produce plots.

## Data Availability

The datasets used and/or analysed during the current study are available from the corresponding author upon reasonable request.
